# Exploring the significance of medical humanities in shaping internship performance: insights from curriculum categories

**DOI:** 10.1080/10872981.2024.2444282

**Published:** 2025-01-25

**Authors:** Chao Ting Chen, Anna Y.Q. Huang, Po-Hsun Hou, Ji-Yang Lin, His-Han Chen, Shiau-Shian Huang, Stephen J. H. Yang

**Affiliations:** a Department of Medical Education, Taipei Veterans General Hospital, Taipei, Taiwan; b Computer Science and Information Engineering, National Central University, Taoyuan, Taiwan; c Department of psychiatry, Taichung Veterans General Hospital, Taichung, Taiwan; d Post-Baccalaureate Medicine, National Chung Hsing University, Taichung, Taiwan; e Department of Psychiatry, Yang Ji Mental Hospital, Keelung, Taiwan; f College of Medicine, National Yang Ming Chiao Tung University, Taipei, Taiwan; g School of Public Health, National Defense Medical Center, Taipei, Taiwan; h Nankung psychiatric Hospital, Keelung, Taiwan

**Keywords:** Medical humanities, medical education, internship performance, machine learning regression, multiple logistic regression

## Abstract

**Background:**

Medical Humanities (MH) curricula integrate humanities disciplines into medical education to nurture essential qualities in future physicians. However, the impact of MH on clinical competencies during formative training phases remains underexplored. This study aimed to determine the influence of MH curricula on internship performance.

**Methods:**

The academic records of 1364 medical students across 8 years of admission cohorts were analyzed. Performance in basic sciences, clinical skills, MH, and internship rotations were investigated, including the subgroup analysis of MH curricula. Ten-fold cross-validation machine learning models (support vector machines, logistic regression, random forest) were performed to predict the internship grades. In addition, multiple variables regression was done to know the independent impact of MH on internship grades.

**Results:**

MH showed the important roles in predicting internship performance in the machine learning model, with substantially reduced predictive accuracy after excluding MH variables (e.g. Area Under the Curve (AUC) declining from 0.781 to 0.742 in logistic regression). Multiple variables regression revealed that MH, after controlling for the scores of other subjects, has the highest odds ratio (OR: 1.29, *p* < 0.0001) on internship grades. MH explained 29.49% of the variance in internship grades as the primary variable in stepwise regression. In the subgroup analysis of MH curricula, Medical Sociology and Cultural Studies, as well as Communication Skills and Interpersonal Relationships, stood out with AUC values of 0.710 and 0.705, respectively, under logistic regression.

**Conclusion:**

MH had the strongest predictive association with clinical competence during formative internship training, beyond basic medical sciences. Integrating humanities merits greater prioritization in medical curricula to nurture skilled, compassionate physicians. Further research should investigate the longitudinal impacts of humanities engagement.

## Introduction

In 1984, physician Eric Cassell recognized the expanding influence of humanities beyond bioethics in the realm of disease and health, emphasizing the symbiotic relationship between medicine and humanities. This intersection provided a fertile ground for exploring philosophical, historical, and literary aspects [1]. Medical humanities (MH), an interdisciplinary field that emerged in the United States during the 1960s, aimed to revive the neglected connection between medicine and the arts [2]. Medical humanities curricula (MHC) integrate various disciplines such as literature, arts, philosophy, ethics, history, theology, anthropology, psychology, and sociology, enriching the educational experience for aspiring healthcare professionals [3,4]. The term ‘MH’ can be categorized into four main rationales: intrinsic, instrumental, critical, and epistemological [5]. These rationales encompass incorporating a humanistic perspective into the curriculum, emphasizing practical knowledge and skills relevant to clinical practice, critically analyzing educational and health practices, and recognizing the fundamental role of humanities in medical education and practice [6–10]. By embracing these subjects, students develop a comprehensive understanding of the multifaceted dimensions intertwined with the practice of medicine.

Traditionally, medical education prioritized scientific knowledge and technical expertise, aiming to enhance diagnostic accuracy and treatment outcomes through structured curricula [11–13]. However, this approach often resulted in burnout syndrome due to the heavy reliance on memorization [14]. Insufficient time for reflection further fostered a dogmatic approach to medical practice [15]. In recent years, the significance of MHC has been acknowledged as an integral part of comprehensive medical training. MHC play a pivotal role in nurturing essential qualities beyond medical procedures and treatments. They foster critical thinking, cultural sensitivity, ethical reasoning, and the ability to navigate complex ethical dilemmas. Notably, studies have shown that systematic observation of artwork can enhance the observational skills of medical students and doctors [16]. These curricula also provide a broader perspective on healthcare by examining historical and societal contexts. They encourage students to explore the diverse experiences of patients, healthcare providers, and communities, cultivating empathy and a deeper understanding of the human condition. Neglecting a humanistic approach in healthcare can lead to a decline in empathy and compassion during clinical encounters, affecting diagnostic precision, patient satisfaction, and treatment adherence, ultimately compromising the effectiveness and quality of care [17].

Integrating MH into the medical curriculum not only fosters personal and professional growth among students but also holds broader implications for the healthcare system. Physicians who have a strong grounding in MH bring forth a multitude of benefits, including enhanced empathy, cultural awareness, observational skills, teamwork, reasoning abilities, listening skills, self-reflection, communication skills, and reduced stress [18,19]. However, a fundamental contradiction exists within modern medical education. While students are introduced to components of MH during the preclinical years to cultivate empathy, compassion, and a patient-centered approach, these values are often overshadowed by a contrasting culture of performance, productivity, and the pressures of efficiency [20]. The necessity of incorporating MH into the curriculum has been the subject of comprehensive discussion. Opponents of compulsory integration of MHC into the curriculum often argue that MH are not as essential in medical education and may potentially detract from students’ focus on medical coursework [21,22]. This viewpoint may indicate that many students perceive MH as separate from their medical knowledge.

Despite evidence suggesting that a positive learning experience in MH enhances future performance, studies have not demonstrated a direct correlation between the quantity of MHC and subsequent academic achievement [23]. Consequently, there is a paucity of research specifically examining the impact of MHC on clinical and internship performance. In Taiwan, internship performance is evaluated through a multifaceted system that includes both written assessments on medical knowledge and practical evaluations. These evaluations incorporate 360-degree peer assessments from interns, residents, and nursing staff, as well as in some departments, patient feedback. Additionally, attending physicians assess students in outpatient clinics based on six core competencies: medical knowledge, communication skills, professionalism, teamwork, learning and growth, and patient care. Through these core competencies and interactions with patients, important attributes such as cooperation and empathy can also be evaluated. These qualities are indirectly assessed within the comprehensive framework, ensuring a well-rounded evaluation of clinical and interpersonal skills. Hence, this study has three research objectives: 1. To utilize MH grades to predict medical students’ performance during hospital internships. 2. To explore the independent impact of MHC grades on internship performance. 3. Finally, if the predictive role of MH on future performance is affirmed, this study aims to investigate which subfield of MH plays the most crucial role.

## Material and methods

### Study participants and data collection

For this cohort study, medical students from the National Yang-Ming University School of Medicine were included, covering eight different years of enrollment cohorts from 2011 to 2019. Each medical student’s academic performance was collected based on the 6 to 7 years of their university education. Most students were admitted based on their performance in the General Scholastic Ability Test (GSAT) and Advanced Subjects Test (AST), which are high-stakes college entrance exams for high-school seniors conducted annually in January and July in Taiwan. Demographic information, including age, gender, residential area, cumulative Grade Point Average (GPA), and course grades, was obtained from the registrar’s office. This study was conducted following the approval of the Institutional Review Board (IRB) at Taipei Veterans General Hospital, under the approval number 2023–08-008AC. The research was conducted in compliance with the ethical guidelines set forth by the IRB. The IRB approval ensures that all aspects of participant privacy, data confidentiality, and ethical treatment were maintained throughout the study.

### Categorizing the medical curriculum

In Taiwan, the medical program follows a structured curriculum that consists of a four-year preclinical phase, which includes 2 years of liberal education and basic medical sciences, followed by a two-year clinical phase. This is then closely followed by a three-year or two-year clinical phase, known as clerkship or internship. In the Taiwanese medical education system, students in clerkships are equivalent to third or fourth-year medical students undergoing clinical training in four-year graduate medical programs in Western countries. For this analysis, the medical education curriculum was categorized into four main groups according to the course content and course sequence. The first category of interest was MH, which encompassed a range of topics including medical ethics and philosophies of the life, medical sociology and cultural studies, communication skills and interpersonal relationships, psychology and mental health, integration of humanities and medicine, and others. The second category focused on basic medical sciences, which encompassed subjects such as anatomy, biochemistry, microbiology, embryology, pathology, physiology, and pharmacology. The third category centered on clinical education, which occurred after the completion of basic medical sciences and involved the exploration of various medical specialties. These curricula included Internal Medicine, Surgery, Obstetrics and Gynecology, Pediatrics, Radiology, Ophthalmology, Dermatology, Psychiatry, and more. Finally, the last category pertained to internship performance, which involved assessing the grades obtained by medical students during their 1 to 2 years of hospital-based rotations prior to graduation. By analyzing these four distinct curriculum categories, this study aimed to gain insights into their respective impacts on medical education and training in Taiwan.

### Data transformation, and variable selection

To predict the influence of different curriculum categories on internship performance by machine learning, we transformed the students’ internship grades from continuous variables to binary variables. Students with internship grades surpassing the median grade of all students were categorized separately from those below the median. We organized the data and removed curricula with fewer student selections, such as Traditional Chinese Medicine, to ensure data quality. Additionally, we explored various independent variables beyond the curriculum categories. This included gender, age at enrollment, type of admission examination method, repeater status, and the number of failed subjects. Through this rigorous approach, we sought to gain insights into the relationship between curriculum categories, independent variables, and internship performance, enabling a more comprehensive understanding of the factors influencing the performance of medical students during their internship.

### Statistical analysis and model selection

**Figure 1.**
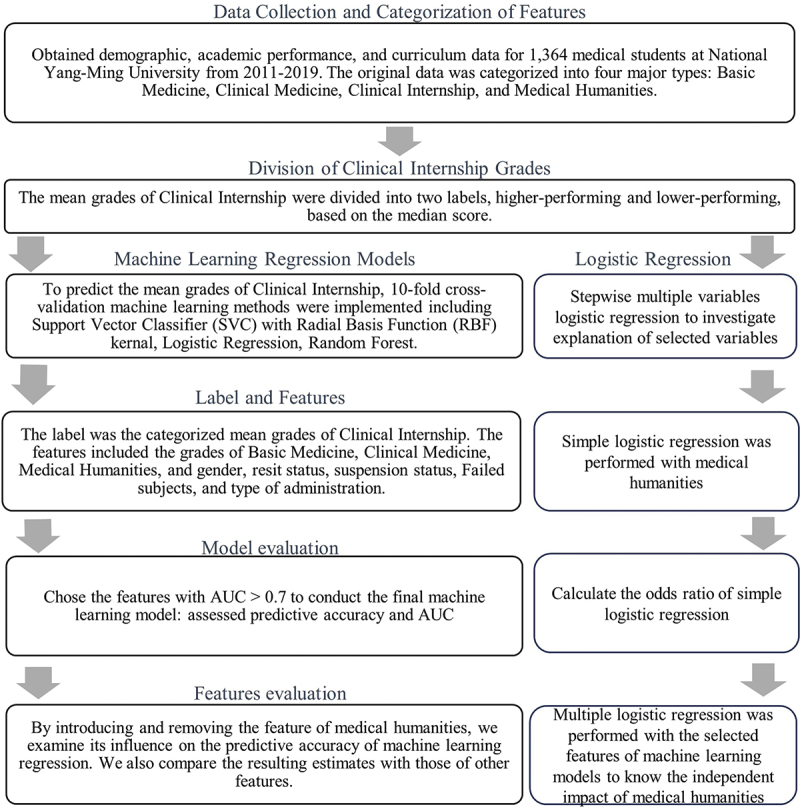
The flow chart of the analytical method.

In this study, we conducted statistical analysis on the four medical domains mentioned above, including MH, basic medical sciences, clinical education, and internship performance. The overall workflow of data collection, categorization, and analysis methods is summarized in Figure 1. The grades of the internship performance served as labels (outcome variable), while the other three domains were used as features (predictive features). Each domain was further divided into subcategories based on the curriculum. Additionally, apart from academic performance, we included additional feature: gender, admission method, repetition of curricula, and whether the students were admission-exam retakers.

For the first aim of predicting students’ learning performance, we utilized three widely recognized machine learning classification models: logistic regression, support vector classification (SVC) with a radial basis function (RBF) kernel, and random forest (RF). These models were selected based on their established effectiveness in handling classification tasks [24]. Logistic regression is a powerful and interpretable model commonly used for binary classification problems. Its simplicity and computational efficiency allow it to handle datasets effectively without overfitting, while still offering clear interpretability through its regression coefficients. SVC with RBF kernel was chosen due to its ability to capture complex, non-linear relationships in the data by finding an optimal hyperplane that separates different classes. SVM models, particularly those using the RBF kernel, have consistently shown strong predictive performance across various datasets, making them a robust choice for classification tasks [24]. Finally, random forest (RF), an ensemble model that combines multiple decision trees, was included due to its high accuracy and ability to handle both linear and non-linear data. Random forest is particularly effective at reducing overfitting by averaging the results of many decision trees and has been shown to be one of the top-performing classifiers in large-scale comparisons [24]. Four metrics, including accuracy, precision, recall, and the Area Under the Curve (AUC) were calculated.

Ten-fold cross-validation method was used to measure the performance of production models based on different machine learning techniques [25]. Onefold was chosen as the test set, while the remaining ninefolds were used as the training set. This process was repeated 10 times, and the results of the 10 tests were averaged to obtain an overall performance evaluation of the model [26]. The evaluation of these models relied on the AUC metric, with a threshold set at >0.7, to discern the significant impact of curriculum categories. Items with an AUC > 0.7 were individually selected as predictor variables, and the final prediction model was re-run accordingly. By iteratively adding and removing the MHC, we aimed to ascertain its importance in the predictive model for medical clinical internships. Furthermore, we sought to explore any potential confounding factors beyond variables with an AUC > 0.7. Through descriptive statistical analysis, significant differences were identified between the two groups in the original grouping. To ensure the accuracy of subsequent regression models, adjustments were also made and established regression models with machine learning.

For the second aim, we employed both simple regression and multivariate regression. By controlling for the influence of other factors, we assessed the independent impact of MH. We conducted simple logistic regression and multiple logistic regression by SAS 9.4. In conducting multiple logistic regression, we utilized significant factors obtained from the machine learning model with AUC > 0.7 to assess the impact of MH within this subset. We also conducted stepwise logistic regression in SAS to compare feature selection and contributions from machine learning.

For the third aim, we conducted subgroup analysis using machine learning models to explore the impact of the six curricula within MH, including Medical Ethics and Philosophies of Life, Medical Sociology and Cultural Studies, Communication Skills and Interpersonal Relationships, Psychology and Mental Health, Integration of Humanities and Medicine, and Others, on clinical internship performance when MH emerged as a significant factor.

## Results

A total of 1,364 students were included in this study. The students were stratified into two groups based on their MH performance, with one group scoring above the median and the other group scoring below the median. Both groups consisted of 682 students separately. Statistical tests revealed significant disparities between the two groups in terms of gender, admission type, admission-exam retaker, and the number of failed subjects. Specifically, the above median humanities group had a significantly higher proportion of female students compared to the below median group (23.46% vs 11.44%, *p* < 0.0001). The above median humanities group also had fewer students admitted through the AST than the below median group (20.53% vs 29.33%, *p* < 0.0001). Nevertheless, the above median humanities group exhibited a lower percentage of students with failed subjects in comparison to the below median humanities group (46.41% vs. 32.48%, *p* < 0.0001). (Table 1)

**Table 1.** Comparing the demographic and academic characteristics of students with better and poorer medical humanities performance (above and below median score of medical humanities).

Variable	Above median group		Below median group		*P* value
	N	%	N	%	
Total	682	50	682	50	
Female	320	23.46	156	11.44	<0.0001
Admission-exam retakers	140	10.26	191	14.00	<0.01
Experiencing suspensions from studies	18	1.32	26	1.19	0.220
Never having failed a subject	633	46.41	443	32.48	<0.0001
Admission by AST ^[a]^	280	20.53	400	29.33	<0.0001

^a^AST: Advanced Subjects Test, University Entrance Examination in Taiwan for High School Students, Including Medical Programs.

The predictive capabilities of Support Vector Classification (SVC), Logistic Regression, and Random Forest machine learning techniques were evaluated for their efficacy in forecasting internship outcomes. This assessment was conducted both with (Table 2a) and without (Table 2b) the inclusion of MH variables. In models that integrated MH variables, Logistic Regression showcased the most favorable overall performance. It achieved an accuracy of 0.689 and an AUC of 0.781. Similarly, SVC exhibited promising predictive validity, attaining an accuracy of 0.688 and an AUC of 0.748. In contrast, Random Forest produced relatively less impressive results, with metrics like Accuracy of 0.66. Notably, Random Forest demonstrated the lowest AUC among the three methods, with a value of 0.725.

**Table 2.** The machine learning prediction model of impact of medical humanities on internship performance.

	Accuracy	Precision	Recall	AUC
(a) Construction of machine learning models using subjects with AUC > 0.7				
SVC with RBF	0.688	0.674	0.677	0.748
Logistic Regression	0.689	0.678	0.680	0.781
Random Forest	0.660	0.647	0.653	0.725
(b) Construction of machine learning models using subjects with AUC > 0.7 excluding medical humanities				
	Accuracy	Precision	Recall	AUC
SVC with RBF	0.681	0.666	0.667	0.698
Logistic Regression	0.674	0.658	0.656	0.742
Random Forest	0.628	0.599	0.603	0.660
(c) Construction of machine learning models using medical humanities only				
	Accuracy	Precision	Recall	AUC
SVC with RBF	0.641	0.662	0.677	0.756
Logistic Regression	0.641	0.651	0.675	0.759
Random Forest	0.562	0.572	0.585	0.645
(d) Construction of machine learning models using subjects with AUC > 0.7 and demographic variables (gender, Never having failed a subject, Admission by AST)				
	Accuracy	Precision	Recall	AUC
SVC with RBF	0.693	0.683	0.683	0.760
Logistic Regression	0.662	0.652	0.667	0.739
Random Forest	0.675	0.661	0.667	0.751

SVC: Support Vector Classification, RBF: Radial Basis Function, AUC: Area Under the Curve.

After removing the MH variables, the predictive performance of all three modeling approaches showed a decline. This highlights the substantial predictive contribution provided by the humanities. While Logistic Regression retained its position as the primary method, its Accuracy decreased to 0.67. For SVC, the Accuracy dropped to 0.681. The most substantial decrease was observed in the case of Random Forest, where the Accuracy dropped significantly to 0.628.

When considering MH alone (Table 2c), Logistic Regression and SVC with RBF stand out, achieving an Accuracy of 0.641 and an AUC of 0.759 and 0.756, respectively. However, Random Forest, in this context, demonstrates comparatively lower predictive validity, with an Accuracy of 0.562 and an AUC of 0.645. To explore the confounding factor, we integrated variable adjustments based on descriptive statistics, including gender, absence of academic failure, and admission via AST to the original machine learning model using subjects with ACU > 0.7. We observed a decrease in the AUC value from 0.781 to 0.739, as well as reductions in accuracy from 0.689 to 0.662 and precision from 0.678 to 0.652 (Table 2d).

The results obtained from the traditional logistic regression emphasized the substantial link between MH and internship performance, which stands out in comparison to the impact of basic sciences. When solely considered as a predictor (Table 3a), MH retained a noteworthy estimate of 0.354, indicating its robust predictive validity. In the comprehensive model including all predictors (Table 3b), the coefficient estimates for MH displayed the highest value at 0.252. In contrast, the estimated coefficients for biology and biochemical genetics were −0.008, microbiology and immunology were 0.022, anatomy were 0.0462 and for clinical skills training were 0.095.

**Table 3.** Simple and multiple logistic regression analyses examining the influence of medical humanities on internship performance.

	OR	95% confidence limits		*P* value
(a) Simple logistic regression				
Medical Humanities	1.426	1.355	1.500	<0.0001
(b) Multiple logistic regression				
Biology and biochemical genetics	0.992	0.958	1.027	0.639
Microbiology and immunology	1.022	0.979	1.067	0.326
Anatomy	1.047	1.017	1.079	<0.005
Clinical skills	1.100	1.061	1.140	<0.0001
Medical humanities	1.287	1.212	1.366	<0.0001

OR: Odds Ratio.

In the context of stepwise logistic regression model selection (Suplementary Table S1), MH emerged as the initial variable of choice, accounting for 29.49% of the variance. Clinical skills were subsequently identified as the second variable, adding a notable 12.81% to the explained variance. Meanwhile, other variables such as gender and basic sciences (1e) were introduced into the model, yet their contributions to variance were comparatively modest.

Based on the results, we further analyzed the role of six different MHC categories in predicting internship performance using machine learning techniques with Support Vector Classification (SVC), Logistic Regression, and Random Forest (Table 4). In line with the previous analysis of overall MH predicting internship outcomes, Logistic Regression exhibited the most favorable performance for these two specific humanities categories. For ‘Medical Sociology and Cultural Studies’, Logistic Regression achieved an AUC of 0.710, while for ‘Communication Skills and Interpersonal Relationships’, it attained an AUC of 0.705. SVC models also displayed promising predictive validity, with AUCs of 0.706 and 0.678 for the two categories, respectively. In contrast, Random Forest trailed behind, with relatively lower AUC values of 0.662 for ‘Medical Sociology and Cultural Studies’ and 0.641 for ‘Communication Skills and Interpersonal Relationships’.

**Table 4.** The machine learning prediction model of impact of subgroup medical humanities on internship performance.

	Accuracy	Precision	Recall	AUC
(a) Medical ethics and philosophies of the life				
SVC with RBF	0.580	0.599	0.603	0.664
Logistic Regression	0.593	0.606	0.617	0.667
Random Forest	0.560	0.573	0.577	0.589
(b) Medical sociology and cultural studies				
SVC with RBF	0.629	0.628	0.635	0.706
Logistic Regression	0.631	0.620	0.631	0.710
Random Forest	0.623	0.611	0.621	0.662
(c) communication skills and interpersonal relationships				
SVC with RBF	0.673	0.625	0.631	0.678
Logistic Regression	0.678	0.639	0.631	0.705
Random Forest	0.641	0.583	0.581	0.641
(d) Psychology and mental health				
SVC with RBF	0.568	0.558	0.571	0.606
Logistic Regression	0.541	0.556	0.568	0.637
Random Forest	0.577	0.541	0.554	0.587
(e) Integration of humanities and medicine				
SVC with RBF	0.569	0.586	0.595	0.655
Logistic Regression	0.577	0.591	0.600	0.664
Random Forest	0.574	0.571	0.579	0.603
(f) Others				
SVC with RBF	0.573	0.495	0.526	0.552
Logistic Regression	0.554	0.515	0.512	0.512
Random Forest	0.594	0.568	0.548	0.617

SVC: Support Vector Classification, RBF: Radial Basis Function.

## Discussion

This study demonstrated that MHC play an important role in the association with the internship performance of medical students. The analysis of four curriculum categories (basic sciences, clinical skills, internship performance, and humanities) for 1,364 students over 8 years’ cohort study provided evidence that MHC had the strongest predictive association with internship grades in machine learning models. Both support vector machines and logistic regression showed reduced predictive accuracy when MH variables were excluded from the models. For instance, the AUC declined from 0.781 to 0.742 in logistic regression after omitting humanities. The observed decrease in predictive performance suggests that gender, failing a subject, and admission method may indeed serve as confounding factors, influencing the relationship between predictor variables and the outcome of interest. Further investigation may be warranted to elucidate the specific mechanisms through which these confounding factors impact predictive performance and to refine model adjustments accordingly.

The regression analysis reinforced the significant association between MH and internship performance. The logistic regression coefficients further emphasized the predominance of MH in relation to basic sciences or clinical skills. After multivariable adjusting, the estimated coefficient for humanities was 0.252, considerably higher than the other four features including Biology and Biochemical Genetics, Microbiology and Immunology, Anatomy, and Clinical Skills Training. When evaluated as the sole predictor variable, MH still demonstrated respectable predictive validity. This indicates that humanities contribute to a substantial proportion of the explainable variance in internship outcomes. The stepwise regression provided additional confirmation, as humanities emerged as the foremost variable, explaining 29.49% of the variance in internship grades. Clinical skills explained only 12.81% of additional variance after humanities were incorporated. Taken together, these findings spotlight the integral role of MH in associating the development of clinical competencies during the formative internship period.

The significant role of MHC in contributing to the development of clinical competencies during medical students’ internships is underscored by our study findings. Specifically, the curriculum focusing on Medical Sociology and Cultural Studies, alongside that of communication skills and interpersonal relationships, emerges as particularly impactful.

Integrating medical sociology into medical education offers a comprehensive approach to training future healthcare professionals [27]. The curriculum provides insights into holistic healthcare models, Taiwan’s medical culture evolution, and community-based care systems. This approach not only imparts theoretical knowledge but also facilitates experiential learning through volunteer activities, enabling students to interact with diverse communities firsthand. Such immersive experiences enhance students’ clinical competencies by deepening their understanding of sociological aspects of healthcare and fostering essential communication skills rooted in mutual understanding [28,29]. Moreover, the significance of training medical students in clinical communication skills cannot be overstated. Effective communication is considered to be a core competency for medical practitioners, playing a vital role in healthcare settings [30,31]. Our study reinforces the importance of MH in enhancing clinical competencies, with a notable emphasis on Medical Sociology & Cultural Studies and Communication Skills & Interpersonal Relationships.

Some limitations should be contemplated when interpreting the study findings. The single-institution design reduces the generalizability of the results. Incorporating students from multiple medical schools could provide more representative insights into the benefits of humanities. Additionally, the observational methodology cannot definitively ascertain causality between humanities exposure and internship outcomes. Experimental designs that manipulate humanities exposure could better determine its direct effects. Experimental studies which directly manipulate the amount of humanities curriculum through random assignment could better isolate its causal effects. Furthermore, self-selection bias may be inherent, as students choose to enroll in humanities electives based on pre-existing interests and aptitudes. However, the design of the academic system, which forces each student to take many MHC, will reduce this bias. Finally, the study was limited to academic performance indicators like GPA. Expanding the outcome measures to include qualities such as empathy, teamwork and communication ability could reveal additional humanities benefits beyond scholastic achievement alone.

## Conclusions

In summary, the study findings advocate for integrating humanities as a fundamental component within medical education, given its pivotal role in nurturing the clinical competencies of future physicians. Humanities curricula cultivate multifaceted skills in observation, critical thinking, ethical reasoning, self-reflection and communication, which translate to superior performance during the demanding clinical rotations of internship. As such, MH merit greater emphasis and resources in medical school curricula worldwide, to foster the professional development of compassionate, well-rounded physicians. Further research should build on these results by evaluating longitudinal outcomes, expanding to varied institutions, utilizing experimental methods, and assessing a wider array of competencies enhanced through engagement with the humanities.

## Supplementary Material

Supplementary_0924_medical educaiton online.docx

## Data sharing statement

The data supporting the findings of this study are available on request from the corresponding author. The data are not publicly available due to privacy or ethical restrictions.

## References

[1] Wailoo K. Patients are humans too: the emergence of medical humanities. Daedalus-Us. 2022;151(3):194–9. doi: 10.1162/daed_a_01938

[2] Evans HM, Greaves DA. Ten years of medical humanities: a decade in the life of a journal and a discipline. Med Humanit. 2010;36(2):66–68. doi: 10.1136/jmh.2010.005603 21393283 PMC3779829

[3] Kirklin D. The centre for medical humanities, royal free and University College Medical School, London, England. Acad Med. 2003;78(10):1048–1053. doi: 10.1097/00001888-200310000-00023 14534108

[4] Shapiro J, Coulehan J, Wear D, et al. Medical humanities and their discontents: definitions, critiques, and implications. Academic Med. 2009;84(2):192–198. doi: 10.1097/ACM.0b013e3181938bca 19174663

[5] Hoang BL, Monrouxe LV, Chen KS, et al. Medical humanities education and its influence on students’ outcomes in Taiwan: a systematic review. Front Med-Lausanne. 2022;9. doi: 10.3389/fmed.2022.857488 PMC9150274 35652071

[6] Macnaughton J. The humanities in medical education: context, outcomes and structures. Med Humanit. 2000;26(1):23–30. doi: 10.1136/mh.26.1.23 12484317

[7] Macneill PU. The arts and medicine: a challenging relationship. Med Humanit. 2011;37(2):85–90. doi: 10.1136/medhum-2011-010044 22114348

[8] Wershof Schwartz A, Abramson JS, Wojnowich I, et al. Evaluating the impact of the humanities in medical education. Mt Sinai J Med. 2009;76(4):372–380. doi: 10.1002/msj.20126 19642151

[9] Chiavaroli N. Knowing how we know: an epistemological rationale for the medical humanities. Med Educ. 2017;51(1):13–21. doi: 10.1111/medu.13147 27981654

[10] Boudreau JD, Fuks A. The humanities in medical education: ways of knowing, doing and being. J Med Humanit. 2015;36(4):321–336. doi: 10.1007/s10912-014-9285-5 24711151

[11] Buja LM. Medical education today: all that glitters is not gold. BMC Med Educ. 2019;19(1):110. doi: 10.1186/s12909-019-1535-9 30991988 PMC6469033

[12] Kuper A, Veinot P, Leavitt J, et al. Epistemology, culture, justice and power: non-bioscientific knowledge for medical training. Med Educ. 2017;51(2):158–173. doi: 10.1111/medu.13115 27862175

[13] Kollmer Horton ME. The orphan child: humanities in modern medical education. Philos Ethics Humanit Med. 2019;14(1):1. doi: 10.1186/s13010-018-0067-y 30616581 PMC6322292

[14] Jennings ML. Medical student burnout: interdisciplinary exploration and analysis. J Med Humanit. 2009;30(4):253–269. doi: 10.1007/s10912-009-9093-5 19865808

[15] Kirklin D, Duncan J, McBride S, et al. A cluster design controlled trial of arts-based observational skills training in primary care. Med Educ. 2007;41(4):395–401. doi: 10.1111/j.1365-2929.2007.02711.x 17430285

[16] Gordon J. Medical humanities: to cure sometimes, to relieve often, to comfort always. Med J Aust. 2005;182(1):5–8. doi: 10.5694/j.1326-5377.2005.tb06543.x 15651937

[17] Carr SE, Noya F, Phillips B, et al. Health humanities curriculum and evaluation in health professions education: a scoping review. BMC Med Educ. 2021;21(1):568. doi: 10.1186/s12909-021-03002-1 34753482 PMC8579562

[18] Yang KT, Lin CC, Chang LY. A program to interest medical students in Changhua, Taiwan in the incorporation of visual arts in medicine. Educ Health (Abingdon). 2011;24(3):563. doi: 10.4103/1357-6283.101421 22267351

[19] Liao HC, Wang YH. The application of heterogeneous cluster grouping to reflective writing for medical humanities literature study to enhance students’ empathy, critical thinking, and reflective writing. BMC Med Educ. 2016;16(1):234. doi: 10.1186/s12909-016-0758-2 27590047 PMC5010711

[20] Assing Hvidt E, Ulso A, Thorngreen CV, et al. Weak inclusion of the medical humanities in medical education: a qualitative study among Danish medical students. BMC Med Educ. 2022;22(1):660. doi: 10.1186/s12909-022-03723-x 36064397 PMC9442995

[21] Blease C. In defence of utility: the medical humanities and medical education. Med Humanit. 2016;42(2):103–108. doi: 10.1136/medhum-2015-010827 26842744

[22] Petrou L, Mittelman E, Osibona O, et al. The role of humanities in the medical curriculum: medical students’ perspectives. BMC Med Educ. 2021;21(1):179. doi: 10.1186/s12909-021-02555-5 33761941 PMC7992827

[23] Huang SS, Ho CC, Chu YR, et al. The quantified analysis of the correlation between medical humanities curriculums and medical students’ performance. BMC Med Educ. 2023;23(1):571. doi: 10.1186/s12909-023-04073-y 37568113 PMC10422819

[24] Fernandez-Delgado M, Cernadas E, Barro S, et al. Do we need hundreds of classifiers to solve real world classification problems? J Mach Learn Res. 2014;15:3133–3181.

[25] Guyon I, Saffari A, Dror G, et al. Model selection: beyond the bayesian/frequentist divide. J Mach Learn Res. 2010;11(1):61–87. doi:10.1515/9781782388678-003

[26] Anguita D, Ghelardoni L, Ghio A, Ridella S, editors. The’k’in K-fold cross validation. ESANN. Netherlands: i6doc; 2012, p. 441–446.

[27] Namazi H, Aramesh K, Larijani B. The doctor-patient relationship: toward a conceptual re-examination. J Med Ethics Hist Med. 2016;9:10. 27957287 PMC5149463

[28] Alsuwaidi L, Powell L, Alhashmi D, et al. Volunteering among pre-clinical medical students: study of its association with academic performance using institutional data. MedEdpublish (2016). 2022;12:24. doi: 10.12688/mep.19105.2 36168531 PMC9370086

[29] Blue AV, Geesey ME, Sheridan ME, et al. Performance outcomes associated with medical school community service. Acad Med. 2006;81(10 Suppl):S79–82. doi: 10.1097/00001888-200610001-00020 17001142

[30] Moezzi M, Rasekh S, Zare E, et al. Evaluating clinical communication skills of medical students, assistants, and professors. BMC Med Educ. 2024;24(1):19. doi: 10.1186/s12909-023-05015-4 38172832 PMC10765785

[31] Choudhary A, Gupta V. Teaching communications skills to medical students: introducing the fine art of medical practice. Int J Appl Basic Med Res. 2015;5(Suppl 1):S41–4. doi: 10.4103/2229-516X.162273 26380210 PMC4552065

