# Supplementary material for: Exploring the significance of medical humanities in shaping internship performance: insights from curriculum categories
Source: Med Educ Online. 2025 Jan 25;30(1):2444282. doi: 10.1080/10872981.2024.2444282 (PMC11770856; doi:10.1080/10872981.2024.2444282)
Supplement: Supplementary_0924_medical educaiton online.docx [file ZMEO_A_2444282_SM1500.docx]

| Supplementary Table 1. Stepwise logistic regression to evaluate the features | | | | | |
| --- | --- | --- | --- | --- | --- |
| Step | Variable selected | Partial  R-square | Model  R-square | F Value | P-value |
| 1 | Medical humanities | 0.295 | 0.295 | 385.98 | <0.0001 |
| 2 | Clinical skills training | 0.128 | 0.423 | 204.64 | <0.0001 |
| 3 | Psychiatry | 0.027 | 0.450 | 45.39 | <0.0001 |
| 4 | Gender | 0.020 | 0.470 | 34.00 | <0.0001 |
| 5 | Emergency sciences | 0.012 | 0.482 | 22.09 | <0.0001 |
| 6 | Cardiovascular Medicine | 0.015 | 0.497 | 27.10 | <0.0001 |
| 7 | Anatomy | 0.011 | 0.508 | 20.01 | <0.0001 |
| 8 | Pathology | 0.009 | 0.517 | 17.28 | <0.0001 |
| 9 | Admission by AST^1^ | 0.005 | 0.522 | 9.87 | <0.01 |
| 10 | Surgery | 0.006 | 0.528 | 11.23 | <0.001 |
| 11 | Nephrology and Urology | 0.004 | 0.532 | 8.04 | <0.01 |

1. AST: Advanced Subjects Test, University Entrance Examination in Taiwan for High School Students, Including Medical Programs

| Supplementary Table 2. Machine learning models of every single course | | | | |
| --- | --- | --- | --- | --- |
| curricula | Accuracy | Precision | Recall | AUC |
| Molecular Biology and Genetics | 0.623 | 0.625 | 0.648 | 0.714 |
| Microbiology and Immunology | 0.648 | 0.635 | 0.643 | 0.711 |
| Embryology | 0.608 | 0.598 | 0.608 | 0.684 |
| Histology and Hematology | 0.638 | 0.621 | 0.634 | 0.699 |
| Anatomy | 0.646 | 0.642 | 0.66 | 0.725 |
| Pharmacology | 0.624 | 0.621 | 0.634 | 0.693 |
| Physiology | 0.617 | 0.597 | 0.609 | 0.675 |
| Pathology | 0.606 | 0.599 | 0.604 | 0.665 |
| Parasitology | 0.611 | 0.595 | 0.611 | 0.67 |
| Neuroanatomy | 0.597 | 0.61 | 0.629 | 0.685 |
| Clinical Skills Training | 0.664 | 0.661 | 0.615 | 0.706 |
| Internal Medicine | 0.528 | 0.584 | 0.585 | 0.652 |
| Cardiology | 0.520 | 0.583 | 0.582 | 0.643 |
| Metabolism and Endocrinology | 0.573 | 0.578 | 0.583 | 0.628 |
| Hematology-Oncology | 0.543 | 0.586 | 0.566 | 0.635 |
| Nephrology and Urology | 0.544 | 0.584 | 0.591 | 0.641 |
| Pulmonology | 0.564 | 0.589 | 0.584 | 0.641 |
| Gastroenterology | 0.608 | 0.606 | 0.613 | 0.661 |
| Surgery | 0.631 | 0.613 | 0.626 | 0.697 |
| Neurology | 0.588 | 0.617 | 0.627 | 0.671 |
| Obstetrics and Gynecology | 0.603 | 0.593 | 0.598 | 0.661 |
| Pediatrics | 0.553 | 0.555 | 0.563 | 0.627 |
| Diagnostic Imaging Radiology | 0.643 | 0.616 | 0.615 | 0.692 |
| Emergency Medicine | 0.653 | 0.625 | 0.626 | 0.682 |
| Rehabilitation Medicine | 0.586 | 0.597 | 0.595 | 0.652 |
| Dermatology | 0.557 | 0.615 | 0.62 | 0.68 |
| Psychiatry | 0.597 | 0.592 | 0.596 | 0.645 |
| Family Medicine | 0.571 | 0.598 | 0.604 | 0.641 |
| Otorhinolaryngology | 0.545 | 0.582 | 0.591 | 0.653 |
| Orthopedics | 0.584 | 0.577 | 0.582 | 0.633 |
| Ophthalmology | 0.64 | 0.602 | 0.604 | 0.66 |
| Anesthesiology | 0.59 | 0.593 | 0.597 | 0.651 |
| Traditional Chinese Medicine | 0.278 | 0.196 | 0.459 | 0.343 |
| Dentistry | 0.573 | 0.615 | 0.629 | 0.675 |
| Forensic Medicine | 0.592 | 0.606 | 0.585 | 0.632 |
| Clinical Skills Training | 0.608 | 0.654 | 0.617 | 0.618 |
| Medical humanities | 0.658 | 0.649 | 0.659 | 0.747 |

**AUC: Area Under the Curve**
